# The Fitting and Furnishing of Wards and Operating Theatres

**Published:** 1906-09-22

**Authors:** 


### THE FITTING AND FURNISHING OF WARDS AND OPERATING THEATRES.

### I.—Sterilising Apparatus.

For surgical instruments, sterilising apparatus used in modern hospitals consist of oblong metal vessels with covers, the vessels being made in nickel, copper, and occasionally electroplate. Inside the vessels are the trays of perforated metal for holding the instruments. They are usually suspended in the sterilising solution with which the outer vessel is charged. The instruments are now all made with metal handles, both to enable them to be left in the solutions without serious damage, and to eliminate all harbour for germs. In some cases it has been found that the cutting edges of the instruments have been attacked in the process of sterilising, but this has been successfully overcome by placing zinc in the sterilising vessels. Heating the solutions is carried out by spirit lamps, by gas ring burners, by steam, and by electricity. The last, where it can be economically applied and is properly fitted, is the most convenient; but it is nearly always too expensive at present rates for electricity. If the hospitals can arrange to be supplied with current from the power companies that are being developed all over the country at the low rates quoted to Parliament, electric heating will be economical, as well as convenient, for a good many purposes. Gas, in the form of the well-known ring burner under the vessel, is perhaps the most convenient and economical method under present conditions. Steam is convenient where there is a steam supply, and it has no smell, but care must be taken to drain the steam pipes that are employed for heating of the water that is formed by condensation.

For sterilising dressings steam is employed almost universally. The dressings are put into metal perforated drums, and these are placed in the heating apparatus, which consists usually of a cylindrical vessel fitted with a double bottom and enclosing jacket. Water is placed in the double bottom, and this being heated becomes steam and passes up through the jacket into the vessel where the drums containing the dressings are. The steam passes freely through the perforations in the drums, through the dressings, and thence to a condenser. For sterilising large things—clothes, sheets, and blankets—the Washington-Lyons steriliser, made by Messrs. Manlove and Alliott, of Nottingham, in which steam is forced into the vessel containing the clothes under pressure is employed. All the large makers of surgical apparatus make the other forms of sterilisers, but a great many of them come from Germany.

### Portable Sanitary Appliances.

These are still made very largely in stoneware, highly glazed, or strong china; but enamelled iron is also rapidly coming to the front, because it is cheaper, stronger, lighter, and the bed-pans can be made in a larger number of parts, this lending itself to cleaning very much more than the china forms. On the other hand, the enamel chips off very quickly, and the aseptic properties of the apparatus are seriously lessened. The writer was informed by the representative of one of the large companies supplying hospitals that the English firms making enamelled iron were not able to make apparatus that did not chip, while the German firms were. There appears to be no difficulty in making enamelled iron utensils to any of the various designs favoured by different doctors. The china vessels are made largely by Messrs. Copeland, of Stoke-on-Trent.

### Operating Tables.

These are made universally of metal, and nearly always in three pieces, each separately controlled by levers or racks, so that the position of the patient can be altered easily and quickly at will. In particular the latest practice arranges that the anæsthetist has a lever or similar appliance at his hand by which he can instantly lower the patient's head if required. Aluminium is finding favour, also copper. At St. Thomas's Hospital the aluminium operating table is heated by two sets of incandescent lamps, similar to those employed in Dowsing and other electric light treatments and in electric radiators. The lamps are fixed under the centre of the table and controlled by switches attached to the legs of the table, one or two sets of lamps being lighted as required. In addition, the strength of the current passing through the lamps is adjusted by a rheostat, the contact switch of which is also within easy reach. The copper table, with leg grooves, at the Middlesex Hospital is by Meyer and Meltzer, of Great Portland Street. At St. Thomas's it may be mentioned that the portable sanitary appliances are placed, when not in use, on a shelf formed by three lines of pipe, through which steam is allowed to pass in cold weather, this arrangement keeping the bed-pans, etc., at a moderate temperature. Operating tables are all arranged on metal tube supports, the legs having india-rubber tyres to the castors and being arranged to move easily in any direction. Many of them are arranged with drainage gutters in the main portion of the table, leading to a pail underneath.

### Lighting the Operating Table.

The methods employed in the leading hospitals are various. In some hospitals the idea is to produce a good general light over the whole room on the lines of sunlight, so that there may be no shadow, wherever the operating surgeon may stand. In others special arrangements are made for illuminating the patient during the operation. Electric lights in some form are always employed, and where a good general illumination is desired, Nernst lamps are becoming the favourite, on account of the whiter light they give. Thirty-two-candle-power incandescent lamps, with the ordinary carbon filament, are also much used, both for illuminating the whole theatre and the operating table and its immediate vicinity. A favourite method is, a number of 32-candle-power lamps are suspended in such a position that a good light is produced all over the table, and the cords of the lamps are arranged to pull on one side, so as to direct the light to any convenient point. Much favour is given, however, to the lamp carried either on the surgeon's head or on an arm swinging near, the lamp being enclosed inside a tube, with a reflector behind and a lens at the end, so that a beam may be directed on the object.

